# Assessing fatigue in childhood cancer survivors: Psychometric properties of the Checklist Individual Strength and the Short Fatigue Questionnaire––a DCCSS LATER study

**DOI:** 10.1002/cam4.4490

**Published:** 2021-12-24

**Authors:** Adriaan Penson, Iris Walraven, Ewald Bronkhorst, Martha A. Grootenhuis, Wim J. E. Tissing, Helena J. H. van der Pal, Andrica C. H. de Vries, Marry M. van den Heuvel‐Eibrink, Sebastian Neggers, Birgitta A. B. Versluys, Marloes Louwerens, Saskia M. F. Pluijm, Nicole Blijlevens, Margriet van der Heiden‐van der Loo, Leontien C. M. Kremer, Eline van Dulmen‐den Broeder, Hans Knoop, Jacqueline Loonen

**Affiliations:** ^1^ Department of Hematology Radboud University Medical Center Nijmegen The Netherlands; ^2^ Department for Health Evidence Radboud University Medical Center Nijmegen The Netherlands; ^3^ Department of Dentistry Radboud Institute for Health Sciences Radboud University Medical Center Nijmegen The Netherlands; ^4^ Department of Psychology Princess Máxima Center for Pediatric Oncology Utrecht The Netherlands; ^5^ Princess Máxima Center for Pediatric Oncology Utrecht The Netherlands; ^6^ Department of Pediatric Oncology/Hematology Beatrix Children’s Hospital/University of Groningen/University Medical Center Groningen Groningen The Netherlands; ^7^ Department of Pediatric Oncology Erasmus Medical Center Rotterdam The Netherlands; ^8^ Department of Pediatric Oncology Erasmus Medical Center Sophia Children’s Hospital Rotterdam The Netherlands; ^9^ Department of Medicine, section Endocrinology Erasmus Medical Center Rotterdam The Netherlands; ^10^ Department of Internal Medicine Leiden University Medical Center Leiden The Netherlands; ^11^ Department Pediatric Oncology Emma Children’s Hospital University of Amsterdam The Netherlands; ^12^ Department of Pediatric Oncology/Hematology Amsterdam University Medical Center Amsterdam The Netherlands; ^13^ Department of Medical Psychology Amsterdam University Medical Centers University of Amsterdam Amsterdam Public health research institute Amsterdam Netherlands

**Keywords:** checklist individual strength, childhood cancer survivors, psychometric properties, severe fatigue, short fatigue questionnaire

## Abstract

**Background:**

Fatigue is often reported by patients with childhood cancer both during and after cancer treatment. Several instruments to measure fatigue exist, although none are specifically validated for use in childhood cancer survivors (CCS). The aim of the current study was to present norm values and psychometric properties of the Checklist Individual Strength (CIS) and Short Fatigue Questionnaire (SFQ) in a nationwide cohort of CCS.

**Methods:**

In total, 2073 participants were included from the Dutch Childhood Cancer Survivor Study (DCCSS) LATER cohort. Normative data, construct validity, structural validity, and internal consistency were calculated for the CIS and SFQ. In addition, reliability and a cutoff score to indicate severe fatigue were determined for the SFQ.

**Results:**

Correlations between CIS/SFQ and vitality measures asking about fatigue were high (>0.8). Correlations between CIS/SFQ and measures of different constructs (sleep, depressive emotions, and role functioning emotional) were moderate (0.4–0.6). Confirmatory factor analysis resulted in a four‐factor solution for the CIS and a one‐factor solution for the SFQ with Cronbach's alpha for each (sub)scale showing good to excellent values (>0.8). Test–retest reliability of the SFQ was adequate (Pearson's correlation = 0.88; ICC = 0.946; weighted Cohen's kappa item scores ranged 0.31–0.50) and a cut‐off score of 18 showed good sensitivity and specificity scores (92.6% and 91.3%, respectively).

**Conclusion:**

The current study shows that the SFQ is a good instrument to screen for severe fatigue in CCS. The CIS can be used as a tool to assess the multiple fatigue dimensions in CCS.

## Graphical abstract

Several instruments to measure fatigue exist, although none are specifically validated for use in childhood cancer survivors (CCS). The current study shows that the SFQ is a good instrument to screen for severe fatigue in CCS and that the CIS can be used as a tool to assess the multiple fatigue dimensions in CCS.

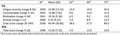

## 1 INTRODUCTION

Cancer‐related fatigue, defined as a distressing, persistent, subjective sense of physical, emotional, and/or cognitive tiredness or exhaustion related to cancer and/or cancer treatment that is not proportional to recent activity and interferes with usual functioning,^1^ is often reported by patients with childhood cancer both during and after (successful) cancer treatment. It was shown to be a debilitating late effect even years after treatment, limiting a person's daily functioning and affecting the quality of life.^2^, ^3^, ^4^ The National Comprehensive Cancer Network (NCCN) recommends to screen all cancer patients for fatigue regularly during and after treatment.^1^ Fatigue is a subjective multidimensional phenomenon best assessed with a questionnaire.^5^ Several instruments to measure fatigue exist, although none are specifically validated for use in adult childhood cancer survivors (CCS).

A frequently used multidimensional questionnaire to measure fatigue is the Checklist Individual Strength (CIS).^6^ It has a total of 20 items using four subscales to distinguish between fatigue severity, concentration problems, reduced motivation, and activity level. The CIS was validated in patients and survivors of adult‐onset cancer,^7^ but not in CCS specifically.

The CIS can be a good instrument to assess the multiple dimensions of fatigue, but to screen for fatigue it is desirable to have a shorter instrument. Guidelines for survivors of adult cancer recommend screening for fatigue using a numerical rating scale (NRS),^1^, ^8^ but this may not be a reliable screening technique in CCS as a single‐item screening instrument was found to not be accurate for identifying cases of clinically significant fatigue in survivors of pediatric brain tumors.^9^ With the current lack of an available adequate screening instrument, the international late effects of childhood cancer guideline harmonization group (IGHG) recommends screening for fatigue performing a short medical history asking about the survivor's feelings of tiredness and exhaustion.^10^ Nonetheless, a systematic measure to indicate whether a person experiences severe fatigue would be preferable. A validated questionnaire with a cut‐off score to indicate severe fatigue could be that measure.

A recent study showed that the Short Fatigue Questionnaire (SFQ),^11^ a short version of the CIS, is an excellent instrument to screen for severe fatigue in the general population and several patient populations, among which survivors of breast cancer and adult hematologic cancer survivors.^12^ With a cut‐off score to indicate severe fatigue,^12^ the SFQ could be an objective screening instrument in CCS.

The SFQ and the CIS are questionnaires that are potentially useful in CCS care. The SFQ as short screening instrument and the CIS as multidimensional fatigue questionnaire have already shown to be valid in multiple patient populations, including cancer patients and survivors of adult‐onset cancer.^7^, ^12^ To test whether previously shown questionnaire properties are also applicable to CCS, validation of both instruments in this patient population is needed. The current study aim was to investigate psychometric properties of the CIS and the SFQ in a nationwide cohort of CCS. Additionally, to indicate how CCS score the CIS and SFQ compared to other populations, norm values are presented.

## 2 METHODS

### 2.1 Participants

Participants were included from the Dutch Childhood Cancer Survivor Study (DCCSS) LATER cohort.^13^ This nationwide cohort CCS, diagnosed before the age of 18 between January 1, 1963 and December 31, 2001 in the Netherlands, was started for a multidisciplinary DCCSS LATER program for CCS late effect care and research. During a clinic visit, which took place in the period 2017–2020, data were collected for the study (details can be found elsewhere^14^). Among other questionnaires (described in detail below), the CIS and SFQ were completed by the participants. A subgroup of the participants completed the SFQ twice within 1 week, one during the clinic visit and a second one digitally at home (second version was part of a questionnaire survey for the whole study cohort which most participants already completed in 2013 except for a small subgroup who were not able to participate in the original survey and were therefore asked to complete it for the current study). All participants gave written informed consent (if aged <16 (*n* = 3), parents gave additional written consent). The study was approved by the Medical Research Ethics Committee of the Amsterdam University Medical Center (registered at toetsingonline.nl, NL34983.018.10).

### 2.2 Fatigue measures

#### 2.2.1 CIS

The Checklist Individual Strength (CIS)^6^ has 20 items (Table S1) and was designed to measure four fatigue dimensions, namely *fatigue severity* (CIS‐fatigue; 8 items), *concentration* (5 items), *motivation* (4 items), and *physical activity level* (3 items). Some items are reversed before scores are added up (Table S1) to calculate the total score, which can range from 20 to 140 with a higher score corresponding to more problematic fatigue. A score of 35 or higher on the subscale *fatigue severity* indicates severe fatigue which was validated in the general Dutch population.^7^

#### 2.2.2 SFQ

The Short Fatigue Questionnaire (SFQ)^11^ consists of four items (identical to four items of the CIS‐fatigue; Table S1) measuring fatigue severity. Three item scores are reversed and then all item scores are added up, resulting in a total score that varies from 4 to 28 with a higher score reflecting more fatigue. The SFQ was validated in the general Dutch population and a cut‐off score of 18 or higher was suggested to indicate severe fatigue.^12^

### 2.3 Other measures

To determine the relationship between the CIS/SFQ and other (fatigue related) measures, two health‐related quality of life questionnaires that include aspects of fatigue (e.g., *vitality*) were completed.

#### 2.3.1 TAAQOL

The TNO and AZL Questionnaire for Adult's Quality of Life (TAAQOL)^15^ has 45 items assessing health‐related quality of life (HRQOL) in 12 domains (*Gross motor function*, *Fine motor function*, *Cognitive function*, *Sleep*, *Pain*, *Social functioning*, *Daily activities*, *Sexuality*, *Vitality*, *Positive emotions*, *Aggressiveness*, *and Depressive emotions*). The subscale *Vitality* contains four items which measure the occurrence of feelings of vitality (Table S1). Scale scores were calculated following instructions described elsewhere^16^ with higher scores indicating good HRQOL. The TAAQOL has been validated in both the general population and in patients with chronic diseases.^15^, ^17^

#### 2.3.2 SF‐36

The Short Form 36 (SF‐36) assesses eight health concepts (*Vitality*, *Physical functioning*, *Bodily pain*, *General health perceptions*, *Role functioning physical*, *Role functioning emotional*, *Social functioning*, *and Mental health*).^18^ The subscale Vitality consists of four items (Table S1), covering feelings of energy and fatigue. Scale scores were calculated following instructions described elsewhere,^19^ so that higher scores indicate better HRQOL. The SF‐36 has been validated in several patient populations, among which cancer patients^20^ and CCS.^21^

The questionnaires were digitally completed during the clinic visit (participants who were not able to visit the clinic or who were not able to complete the questionnaires during the visit were asked to complete a digital or paper versions at home).

### 2.4 Statistical analysis

IBM SPSS (IBM Corp. Released 2017. IBM SPSS Statistics for Windows, Version 25.0. Armonk, NY: IBM Corp) and *R* ^22^ were used to conduct the analyses (all tests with α = 0.05). To examine possible selection bias between study participants and non‐participants (eligible CCS that did not return informed consent or did not complete study questionnaires), groups were compared to sex, decade of birth, childhood cancer diagnosis, decade of diagnosis, treatment with chemotherapy, and /or radiotherapy (yes/no). Cramér's V was calculated to examine effects sizes of potential differences between the groups. Seven hundred and forty‐four persons explicitly refused to participate (see flowchart in Figure S1) and were therefore excluded from this analysis.

#### 2.4.1 Normative data

Mean total scores and percentile scores of the SFQ and CIS (total and subscale scores) were calculated.

#### 2.4.2 Construct validity

Pearson's correlation between the CIS/SFQ and the vitality subscale of the TAAQOL and the SF‐36 was calculated (convergent validity). As both vitality subscales assess symptoms of fatigue, strong correlations (r ≥ 0.7) were expected. Pearson's correlation between the CIS/SFQ and the sleep and depressive emotions subscales of the TAAQOL and the role functioning emotional subscale of the SF‐36 were also calculated (discriminant validity). We assume fatigue to be moderately related with the HRQOL concepts sleep, depressive emotions, and emotional functioning (r between 0.4 and 0.7) because it concerns concepts that have certain overlap, but differ from fatigue as was pointed out by previous studies.^23^, ^24^

#### 2.4.3 Structural validity

Confirmatory factor analysis (CFA) was performed to determine a single‐factor structure for the SFQ (fatigue severity) and a four‐factor structure for the CIS (fatigue severity, concentration, motivation, and physical activity). The structure of both instruments had already been validated in the general (Dutch) population,^7^, ^12^ however has yet to be confirmed in CCS specifically. Maximum likelihood estimation^25^ with direct Oblimin rotation^26^ was performed. Eigenvalues ≥1 were used to identify factors and then factor loadings for each item were calculated (>0.4 was considered good factor loading). Item correlation matrix, the Kaiser–Meyer–Olkin (KMO) test, and the Bartlett's test of sphericity were calculated to test adequacy of the data to perform a factor analysis. Model fit was examined using the root mean square error of approximation (RMSEA), with a value <0.06 as a cut‐off value to indicate good model fit.^27^

#### 2.4.4 Internal consistency

To indicate whether the items of the instruments measure the same underlying constructs, Cronbach's alpha was calculated for the SFQ and the four subscales of the CIS. It is a measure between 0 and 1 indicating how items hold together in a scale where >0.9 is seen as excellent, >0.8 as good, >0.7 as acceptable, and <0.6 as poor internal consistency.

#### 2.4.5 Reliability

To determine test–retest reliability of the SFQ, data from a subgroup of the participants (n=90) who completed the SFQ twice within 1 week, were analyzed in three ways (following Bruton, Conway & Holgate who proposed that a combination of approaches is more likely to give a true picture of the instrument's reliability^28^). Pearson's correlation and intraclass correlation coefficient (ICC)^29^ between the two measurement moments were calculated for total scores and weighted Cohen's Kappa (Kw)^30^ for all individual items of the SFQ. To calculate the ICC, a two‐way random effects, absolute agreement, single measurement model was used.^31^ The CIS was only completed once, therefore its test–retest reliability could not be investigated.

#### 2.4.6 Cut‐off score SFQ

To confirm whether the suggested cut‐off score of 18 for the SFQ to determine severe fatigue can also be used in the CCS population, an ROC analysis was performed. True “severe fatigue cases” were determined using the cut‐off score of 35 on the *CIS*‐*fatigue*. Sensitivity (proportion of truly identified severe fatigue cases) and specificity (proportion of truly identified non‐cases) were calculated for a range of possible cut‐off scores for the SFQ (18 ± 2). Youden's index was calculated, a value between 0 and 1 with a higher value suggesting a better cut‐off point.^32^, ^33^ In addition, the positive prediction value (PPV; proportion of severe fatigue cases identified by the SFQ that are true cases) and negative prediction value (NPV; proportion of non‐cases identified by the SFQ that are truly non‐cases) were calculated.

If a participant had one or more missing values for the CIS/SFQ items and therefore no subscale score could be determined, the participant was excluded from the analyses of that particular subscale. Table S1 shows the number of participants for each subscale.

## 3 RESULTS

In total, 2073 participants (43.8% of eligible persons) were included in the current study (flowchart in Figure S1). A comparison with non‐participants is shown in Table S3. There are no large differences between the groups (small effect sizes), suggesting no selection bias in the study cohort. Table 1 shows the participant characteristics.

**TABLE 1:** Participant characteristics (*n* = 2073)

Characteristic	No.	%
Sex		
Male	1055	50.9
Female	1018	49.1
Age at participation (years)		
<20	67	3.2
20–29	593	28.7
30–39	770	37.1
≥40	643	31.0
Age at diagnosis (years)		
0–5	972	46.9
5–10	554	26.7
10–15	431	20.8
15–18	116	5.6
Primary childhood cancer diagnosis^a^		
Leukemia	736	35.5
Non‐Hodgkin lymphoma^b^	243	11.7
Hodgkin lymphoma	141	6.8
CNS	192	9.3
Neuroblastoma	124	6.0
Retinoblastoma	11	0.5
Renal tumors	237	11.4
Hepatic tumors	18	0.9
Bone tumors	121	5.8
Soft tissue tumors	146	7.1
Germ cell tumors	72	3.5
Other and unspecified^c^	32	1.5
Period of childhood cancer diagnosis		
1960–1969	31	1.5
1970–1979	284	13.7
1980–1989	640	30.9
>1990	1118	53.9
Childhood cancer treatment^d^		
Surgery only	143	6.9
Chemotherapy, no radiotherapy	1112	53.6
Radiotherapy, no chemotherapy	106	5.1
Radiotherapy and chemotherapy	700	33.8
No treatment or treatment unknown	12	0.6

^a^ Diagnostic groups included all malignancies covered by the third edition of the International Classification of Childhood Cancer (ICCC‐3).

^b^ Includes all morphology codes specified in the ICCC‐3 under lymphomas and reticuloendothelial neoplasms, except for Hodgkin lymphomas.

^c^ Includes all morphology codes specified in the ICC‐3 under other malignant epithelial neoplasms and malignant melanomas and other and unspecified malignant neoplasms.

^d^ Treatment data included primary treatment and all recurrences.

### 3.1 Normative data

Mean total scores and subscale scores for the CIS and SFQ are shown in Table 2. Also, percentile scores (25^th^, 50^th^, and 75^th^) are presented.

**TABLE 2:** Norm values for the CIS and SFQ

	*N* ^a^	Mean (SD)	25^th^	50^th^	75^th^
CIS					
Fatigue severity (range 8–56)	2059	25.89 (13.14)	14.0	24.0	36.0
Concentration (range 5–35)	2055	14.98 (7.82)	9.0	13.0	21.0
Motivation (range 4–28)	2061	10.36 (5.38)	6.0	9.0	14.0
Activity (range 3–21)	2060	9.24 (5.23)	5.0	8.0	13.0
Total score (range 20–140)	2038	60.44 (26.85)	38.0	57.0	80.0
SFQ					
Total score (range 4–28)	1998	13.36 (7.32)	7.0	13.0	19.0

^a^ Sum scores were calculated when all items for that particular scale were completed by the participant.

### 3.2 Construct validity

Pearson's correlations between the total score of the CIS/SFQ and the vitality, sleep, and depressive emotions subscales of the TAAQOL and the vitality and emotional role functioning subscales of the SF‐36 are shown in Table 3. Correlations between CIS/SFQ and the vitality subscales were high (>0.8), indicating good convergent validity. Correlations between CIS/SFQ and the HRQOL domains sleep, depressive emotions, and role functioning emotional were moderate (between 0.4 and 0.6), indicating good discriminant validity.

**TABLE 3:** Pearson's correlations between total scores of the CIS and SFQ and subscales of the TAAQOL and SF‐36

	CIS	SFQ
TAAQOL		
Vitality	−0.857	−0.889
Sleep	−0.471	−0.451
Depressive emotions	−0.574	−0.507
SF−36		
Vitality	−0.841	−0.844
Emotional role functioning	−0.522	−0.461

Note:

All correlations were statistically significant (p < 0.01).

### 3.3 Structural validity

#### 3.3.1 CIS

Item correlations for items of the same subscale ranged 0.51–0.82 and for items of different subscales ranged 0.28–0.78. The KMO test showed a value of 0.96, the Bartlett’s test of sphericity was significant (*p *< 0.001), and the RMSEA was 0.07. CFA resulted in a four‐factor solution with each factor explaining 53.7%, 11.0%, 6.4%, and 5.0% of the variance, respectively (76.1% total variance explained). In Figure S2A, the scree plot is shown confirming a four‐factor solution. The eigenvalues for the four factors and factor loadings of all items (range 0.443–0.925) are shown in Table S2. All items loaded good (>0.4) on their original subscale, with items 14 and 20 loading good on two subscales (*fatigue severity* and *activity* subscale).

#### 3.3.2 SFQ

Item correlations were all >0.7, the KMO test showed a value of 0.84, the Bartlett's test of sphericity was significant (*p *< 0.001), and the RMSEA was 0.13. CFA resulted in a one‐factor solution explaining 81.1% of the variance. In Figure S2B, the scree plot is shown with an eigenvalue of 3.245 confirming a one‐factor solution. Table S2 presents the factor loadings of the items, which were all good (range 0.785–0.939).

### 3.4 Internal consistency

Cronbach's alpha for the subscales *fatigue severity*, *concentration*, *motivation,* and *physical activity level* of the CIS was 0.95, 0.91, 0.85, and 0.91, respectively (alpha for all 20 items was 0.95), indicating good to excellent internal consistency. Cronbach's alpha for the SFQ was 0.92 indicating excellent internal consistency.

### 3.5 Reliability

A total of 90 participants completed the SFQ twice within 1 week. Thirty‐nine participants completed both SFQ questionnaires on the same day and 51 participants completed the second SFQ within a week of the first one (mean number of days between both measurements was 4 days; *n* = 90). Pearson's correlation between total scores of the two SFQ measurements was high (0.88; *p *< 0.001) and the ICC was excellent (0.946; 95%CI: 0.907–0.967). Kw scores for item 1–4 were 0.50, 0.43, 0.31, and 0.34, respectively, (all *p *< 0.01) reflecting fair to moderate item agreement.

### 3.6 Cut‐off score SFQ

ROC analysis showed an area under the curve of 0.974 (95% CI: 0.969–0.980). Sensitivity and specificity of the suggested cut‐off score of 18 (± 2) are presented in Table 4. This table also shows the PPV and NPV. The suggested cut‐off score of 18 had the highest value for the Youden's index (highest combined sensitivity and specificity) and showed good PPV and NPV.

**TABLE 4:** Sensitivity, specificity, positive prediction value (PPV), and negative prediction value (NPV) of several SFQ cut‐off scores

SFQ total score	Sensitivity %	Specificity %	PPV %	NPV %
16	97.0	81.8	68.1	98.6
17	95.4	87.0	74.7	97.9
18	92.6	91.3	81.0	96.9
19	86.1	94.6	86.5	94.4
20	75.9	97.2	91.5	90.9

## 4 DISCUSSION

The aim of the current study was to present norm values and psychometric properties of the SFQ, a four‐item screening instrument for severe fatigue, and the CIS, a multidimensional fatigue questionnaire, in a nationwide cohort of CCS. Results show psychometric properties of the SFQ and CIS to be good in CCS and therefore the SFQ can be used to screen for severe fatigue in this population and the CIS can be used to evaluate the multiple fatigue dimensions.

The IGHG guideline^10^ suggests to screen regularly for severe fatigue, however no screening instrument had yet been validated in CCS. Single‐item screening (with the fatigue thermometer) was shown to not be reliable to indicate clinically significant fatigue in survivors of adolescent brain tumors^9^ suggesting a multiple‐item instrument to be more optimal for screening in CCS. With the lack of a validated screening instrument, it is currently suggested to screen for fatigue by performing a medical history focused on the survivors feelings of tiredness and exhaustion (at every long‐term follow‐up visit). Recommended questions to ask are “do you get tired easily” or “are you too tired or exhausted to enjoy the things you like to do.”^10^ The first of these questions is asked in the SFQ, accompanied by questions asking about exhaustion and fitness level. Looking at the suggestions made in the guideline, the SFQ meets the requirements to screen for fatigue in CCS. The current study showed psychometric properties of the SFQ in CCS to be adequate plus the suggested cut‐off score of 18 to indicate severe fatigue showed the highest combined sensitivity and specificity, in addition to a good PPV and NPV in CCS and can therefore be perfectly used for fatigue surveillance.

The guideline further suggests additional testing with a validated fatigue measure for survivors with an indication for severe fatigue.^10^ The *PedsQL Multidimensional Fatigue Scale* or the *PROMIS Pediatric Fatigue measure* is suggested as both have been validated in CCS.^34^, ^35^ However, psychometric properties presented in those studies are limited. Psychometric properties presented of the *PedsQL* by Robert et al.^34^ are good (Cronbach's alpha of total score and three subscales all ≥0.88), but other psychometric properties remained to be determined. Also, the cohort in which the study was conducted was relatively small (*n* = 64) and did not include all childhood malignancies (only CNS, hematological, lymphoma, and solid tumor cancer diagnoses were included). The study by Hinds et al.^35^ showed the *PROMIS Pediatric Fatigue measure* to be a valid instrument to distinguish different levels of fatigue and that it is feasible for cancer patients and survivor populations to properly complete the questionnaire. However, no psychometric properties of the PROMIS in CCS were presented and the studied cohort only included survivors of leukemia, lymphoma, brain tumors, or solid tumors. The current study was the first to validate the CIS in a nationwide cohort CSS including all childhood malignancies and showed the CIS to be a good instrument to investigate multiple dimensions of fatigue. The structural validity we found in CCS (four‐factor solution) is comparable to what has been reported in the general Dutch population,^7^ a Japanese working population,^36^ a healthy Portuguese population,^37^ and a population of patients with rheumatoid arthritis.^38^ Item 14 (Physically I am in bad shape) and 20 (Physically I feel I am in good shape) had good factor loadings (≥0.4) for both the *fatigue severity* subscale and the *physical activity* subscale meaning these items could be used for both subscales. However, to ensure optimal comparison of subscale scores between different populations, we suggest using the original structure of the CIS (item 14 and 20 in *fatigue severity* subscale). Correlations with the vitality subscales of the SF‐36 and TAAQOL were good (Table 3). A high correlation with these subscales that ask about (life) energy, tiredness, and exhaustion mean that these issues and symptoms of fatigue are reflected in the total score of the CIS and SFQ as well. On the other hand, moderate correlations with the sleep, depressive emotions, and role functioning emotional subscales show that the CIS and SFQ can discriminate well between fatigue and these, often with fatigue interfering, symptoms.

Norm values can help interpreting results. Subscale scores and total norm scores of the CIS were comparable to norm scores of adult‐onset breast cancer and hematological cancer survivors.^7^ Compared to norms of the general Dutch population, CCS score higher on all subscales and the total score of the CIS. As previous literature showed symptoms of fatigue to be more prevalent in CCS compared to controls,^4^, ^39^, ^40^ it was expected that CCS would show higher norm values. Since no large differences in diagnosis and treatment‐related variables between participants and non‐participants were found, we assume norm values of the current CCS study cohort to be generalizable.

A limitation of the current study was the lack of a gold standard for confirming the cut‐off score of 18 for severe fatigue of the SFQ. No validated cut‐off instrument to indicate severe fatigue was yet available in CCS and therefore we used the cut‐off score of the CIS (≥35) as a gold standard in the current study. The current study showed the structure and internal consistency of the items and subscales of the CIS to be good and comparable to populations it is already been widely used in (general population, survivors of adult cancer)^7^ and we therefore believe that the cut‐off score of 35 can be safely used in CCS as well.

To conclude, with a growing population of cancer survivors worldwide and fatigue as a frequently reported late effect, structural screening for clinically significant fatigue will become more and more important. The current study shows the SFQ to be a good instrument to screen for severe fatigue in CCS. Would the SFQ indicate a person to be severely fatigued (total score ≥18), it is suggested to do additional testing and the CIS can then be used as a tool to assess the multiple fatigue dimensions.

## CONFLICT OF INTEREST

The authors declare that they have no competing interests.

## AUTHOR CONTRIBUTIONS

Adriaan Penson: Conceptualization, data curation, formal analysis, investigation, methodology, project administration, validation, visualization, writing ‐ original draft. Iris Walraven: writing ‐ original draft, writing ‐ review and editing. Ewald Bronckhorst: Formal analysis, writing ‐ review and editing. Martha Grootenhuis: Investigation, methodology, resources, writing ‐ review and editing. Wim Tissing: Investigation, methodology, resources, writing ‐ review and editing. Helena van der Pal: Investigation, methodology, resources, writing ‐ review and editing. Andrica de Vries: Investigation, methodology, resources, writing ‐ review and editing. Marry van den Heuvel‐Eibrink: Investigation, methodology, resources, writing ‐ review and editing. Sebastian Neggers: Investigation, methodology, resources, writing ‐ review and editing. Birgitta Versluys: Investigation, methodology, resources, writing ‐ review and editing. Marloes Louwerens: Investigation, methodology, resources, writing ‐ review and editing. Saskia Pluijm: Investigation, methodology, resources, writing ‐ review and editing. Margriet van der Heiden‐van der Loo: data curation, software, writing ‐ review and editing. Nicole Blijlevens: Supervision, writing ‐ review and editing. Eline van Dulmen‐den Broeder: Investigation, methodology, resources, writing ‐ review and editing. Leontien Kremer: Funding acquisition, investigation, methodology, resources, writing ‐ review and editing. Hans Knoop: Conceptualization, methodology, project administration, supervision, writing ‐ original draft, writing ‐ review and editing. Jacqueline Loonen: Conceptualization, methodology, project administration, supervision, funding acquisition, writing ‐ original draft, writing ‐ review and editing.

## Supporting information

Supplementary Material

## Data Availability

The data underlying this article were provided by the DCOG‐LATER consortium under license. Data will be shared upon request to the corresponding author with permission of the DCOG‐LATER consortium.
