# Supplementary material for: Assessing fatigue in childhood cancer survivors: Psychometric properties of the Checklist Individual Strength and the Short Fatigue Questionnaire––a DCCSS LATER study
Source: Cancer Med. 2021 Dec 24;11(4):1172–80. doi: 10.1002/cam4.4490 (PMC8855897; doi:10.1002/cam4.4490)
Supplement: Supplementary file 1 — Supplementary Material [file CAM4-11-1172-s001.docx]

Supplementary material

**Supplemental Figure 1. Flowchart of participant inclusion.**


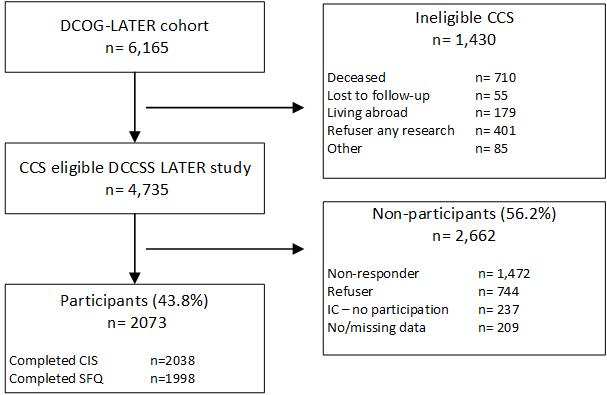


**Supplemental Figure 2. Scree plots of the CIS (A) and SFQ (B).**

**Supplemental Table 1. Items of the fatigue measures**

| **Item** | **Answer options** | **No. (%) ^a^** |
| --- | --- | --- |
| *CIS* |  | 2038 (98.3) |
| Please indicate to what extend the following statements applied to you during the past two weeks |  |  |
| *Subscale fatigue severity* |  | 2059 (99.3) |
| - I feel tired ^c^ | Yes, that is true □ □ □ □ □ □ □ No, that is not true |  |
| - Physically, I feel exhausted ^c^ | “ |  |
| - I feel fit ^b^ | “ |  |
| - I feel weak ^c^ | “ |  |
| - I feel rested ^b^ | “ |  |
| - Physically I am in bad shape ^c^ | “ |  |
| - I tire easily ^c^ | “ |  |
| - Physically I am in good shape ^b^ | “ |  |
| *Subscale concentration* |  | 2055 (99.1) |
| - Thinking requires effort ^c^ | Yes, that is true □ □ □ □ □ □ □ No, that is not true |  |
| - When I am doing something, I can keep my thoughts on it ^b^ | “ |  |
| - I find it easy to concentrate ^b^ | “ |  |
| - It takes a lot of effort to concentrate on things ^c^ | “ |  |
| - My thoughts easily wander ^c^ | “ |  |
| *Subscale motivation* |  | 2061 (99.4) |
| - I feel very active ^b^ | Yes, that is true □ □ □ □ □ □ □ No, that is not true |  |
| - I feel like doing all kinds of nice things ^b^ | “ |  |
| - I have a lot of plans ^b^ | “ |  |
| - I don’t feel like doing anything ^c^ | “ |  |
| *Subscale physical activity level* |  | 2060 (99.4) |
| - Physically I am very active ^b^ | Yes, that is true □ □ □ □ □ □ □ No, that is not true |  |
| - Physically I am little active ^c^ | “ |  |
| - My physical activity level is low ^c^ | “ |  |
|  |  |  |
| SFQ |  | 1998 (96.4) |
| Please indicate to what extend the following statements applied to you during the past two weeks |  |  |
| - I feel tired ^c^ | Yes, that is true □ □ □ □ □ □ □ No, that is not true |  |
| - I tire easily ^c^ | “ |  |
| - I feel fit ^b^ | “ |  |
| - Physically, I feel exhausted ^c^ | “ |  |
|  |  |  |
| *TAAQOL subscale vitality* |  | 1758 (84.8) |
| During the past four weeks, did you feel |  |  |
| - energetic? | □ no □ a little □ some □ a lot |  |
| - tired? | “ |  |
| - fit? | “ |  |
| - exhausted quickly? | “ |  |
|  |  |  |
| *SF-36 subscale vitality* |  | 1716 (82.8) |
| During the past four weeks, did you |  |  |
| - feel full of life? | □ always □ most of the time □ often □ sometimes □ rarely □ never |  |
| - have a lot of energy? | “ |  |
| - feel worn out? | “ |  |
| - feel tired? | “ |  |
|  |  |  |

*^a^ Number of participants that a subscale score could be calculated for.*

*^b^ Normal items scoring (yes, that is true =1, no, that is not true=7)*

*^c^ Reversed items scoring (yes, that is true =7, no, that is not true=1)*

**Supplemental Table 2. Factor loadings of items.**

| **CIS** |  |  |  |  |  |
| --- | --- | --- | --- | --- | --- |
| Items | Factor 1  Fatigue severity | Factor 2  Concentration | Factor 3  Motivation | Factor 4  Activity | Communality |
| Fatigue severity items  1 I feel tired  4 Physically I feel exhausted  6 I feel fit  9 I feel weak  12 I feel rested  14 Physically I am in bad shape  16 I tire easily  20 Physically I feel I am in good shape | 0.925  0.771  0.464  0.443  0.768  0.453  0.799  0.445 | 0.038  0.020  0.019  0.155  0.113  0.011  0.045  0.010 | 0.023  0.062  0.221  0.114  0.058  0.043  0.002  0.035 | 0.073  0.062  0.317  0.166  0.026  0.551  0.072  0.490 | 0.838  0.737  0.762  0.545  0.732  0.698  0.761  0.738 |
| Concentration items  3 Thinking requires effort  8 When I am doing something, I can keep my thoughts on it  11 I find it easy to concentrate  13 It takes a lot of effort to concentrate  19 My thoughts easily wander | 0.236  0.063  0.007  0.004  0.011 | 0.563  0.881  0.898  0.864  0.775 | 0.069  0.008  0.025  0.021  0.014 | 0.033  0.013  0.008  0.015  0.020 | 0.560  0.732  0.817  0.734  0.611 |
| Motivation items  2 I feel very active  5 I feel like doing all kinds of nice things  15 I have a lot of plans  18 I don’t feel like doing anything | 0.263  0.080  0.106  0.112 | 0.001  0.042  0.022  0.140 | 0.444  0.838  0.735  0.623 | 0.240  0.033  0.026  0.010 | 0.655  0.710  0.501  0.617 |
| Activity items  7 I think I do a lot in a day  10 I think I do very little in a day  17 My physical activity level is low | 0.028  0.089  0.065 | 0.009  0.055  0.072 | 0.159  0.006  0.015 | 0.799  0.908  0.794 | 0.773  0.763  0.768 |
| Initial Eigenvalue | 10.748 | 2.194 | 1.281 | 1.006 |  |
| Explained variance (total: 76.1%) | 53.7% | 11.0% | 6.4% | 5.0% |  |
| **SFQ** |  |  |  |  |  |
| Items | Factor 1  Fatigue severity |  |  |  |  |
| 1 I feel tired | 0.939 |  |  |  | 0.882 |
| 2 I tire easily | 0.906 |  |  |  | 0.821 |
| 3 I feel fit | 0.822 |  |  |  | 0.676 |
| 4 Physically I feel exhausted | 0.785 |  |  |  | 0.617 |
| Initial Eigenvalue | 3.245 |  |  |  |  |
| Explained variance | 81.1% |  |  |  |  |

**Supplemental Table 3. Comparison participants vs. non-participants.**

| **Characteristic** | **Participants (n=2073)**  **N (%)** | **Non-participants (n=1918)***  **N (%)** | **ES ^e^** |
| --- | --- | --- | --- |
| **Female sex** | 1018 (49.1) | 736 (38.4) | 0.11 |
| **Decade of birth**  <1960  1960-1969  1970-1979  1980-1989  ≥1990 | 22 (1.1)  166 (8.0)  539 (26.0)  784 (37.8)  562 (27.1) | 20 (1.0)  137 (7.1)  479 (25.0)  745 (38.8)  537 (28.0) | 0.02 |
| **Age at diagnosis**  0-5  5-10  10-15  15-18 | 972 (46.9)  554 (26.7)  431 (20.8)  116 (5.6) | 891 (46.5)  529 (27.6)  376 (19.6)  122 (6.3) | 0.02 |
| **Primary childhood cancer diagnosis ^a^**  Leukemia  Non-Hodgkin lymphoma ^b^  Hodgkin lymphoma  CNS  Neuroblastoma  Retinoblastoma  Renal tumors  Hepatic tumors  Bone tumors  Soft tissue tumors  Germ cell tumors  Other and unspecified ^c^ | 736 (35.5)  243 (11.7)  141 (6.8)  192 (9.3)  124 (6.0)  11 (0.5)  237 (11.4)  18 (0.9)  121 (5.8)  146 (7.0)  72 (3.5)  32 (1.5) | 626 (32.6)  228 (11.9)  142 (7.4)  229 (11.9)  95 (5.0)  13 (0.7)  207 (10.8)  24 (1.3)  100 (5.2)  147 (7.7)  79 (4.1)  28 (1.5) | 0.06 |
| **Childhood cancer treatment ^d^**  Surgery only  Chemotherapy, no radiotherapy  Radiotherapy, no chemotherapy  Radiotherapy and chemotherapy  No treatment/treatment unknown | 143 (6.9)  1112 (53.6)  106 (5.1)  700 (33.8)  12 (0.6) | 232 (12.1)  1098 (57.2)  119 (6.2)  443 (23.1)  26 (1.4) | 0.14 |
| **Recurrence**  No  Yes | 1797 (86.7)  276 (13.3) | 1699 (88.6)  219 (11.4) | 0.03 |

**Refusers (n=744) were excluded from the analysis.*

*^a^ Diagnostic groups included all malignancies covered by the third edition of the International Classification of Childhood Cancer (ICCC-3).*

*^b^ Includes all morphology codes specified in the ICCC-3 under lymphomas and reticuloendothelial neoplasms, except for Hodgkin lymphomas.*

*^c^ Includes all morphology codes specified in the ICC-3 under other malignant epithelial neoplasms and malignant melanomas and other and unspecified malignant neoplasms.*

*^d^ Treatment data included primary treatment and all recurrences.*

*^e^ Effect size, calculated as Cramér’s V (<0.1=little, 0.1=low, 0.3=medium, 0.5=high).*
